# Using Facebook Advertisements for Women’s Health Research: Methodology and Outcomes of an Observational Study

**DOI:** 10.2196/31759

**Published:** 2022-01-12

**Authors:** Deeonna E Farr, Darian A Battle, Marla B Hall

**Affiliations:** 1 Department of Health Education and Promotion College of Health and Human Performance East Carolina University Greenville, NC United States; 2 Department of Public Health Brody School of Medicine East Carolina University Greenville, NC United States

**Keywords:** social media, surveys, questionnaires, advertising, patient selection, methodology, ethnic groups, health research, healthcare, health care, women’s health

## Abstract

**Background:**

Recruitment of diverse populations for health research studies remains a challenge. The COVID-19 pandemic has exacerbated these challenges by limiting in-person recruitment efforts and placing additional demands on potential participants. Social media, through the use of Facebook advertisements, has the potential to address recruitment challenges. However, existing reports are inconsistent with regard to the success of this strategy. Additionally, limited information is available about processes that can be used to increase the diversity of study participants.

**Objective:**

A Qualtrics survey was fielded to ascertain women’s knowledge of and health care experiences related to breast density. This paper describes the process of using Facebook advertisements for recruitment and the effectiveness of various advertisement strategies.

**Methods:**

Facebook advertisements were placed in 2 rounds between June and July 2020. During round 1, multiple combinations of headlines and interest terms were tested to determine the most cost-effective advertisement. The best performing advertisement was used in round 2 in combination with various strategies to enhance the diversity of the survey sample. Advertisement performance, cost, and survey respondent data were collected and examined.

**Results:**

In round 1, a total of 45 advertisements with 5 different headlines were placed, and the average cost per link click for each headline ranged from US $0.12 to US $0.79. Of the 164 women recruited in round 1, in total 91.62% were eligible to complete the survey. Advertisements used during recruitment in round 2 resulted in an average cost per link click of US $0.11. During the second round, 478 women attempted the survey, and 87.44% were eligible to participate. The majority of survey respondents were White (80.41%), over the age of 55 years (63.94%), and highly educated (63.71%).

**Conclusions:**

Facebook advertisements can be used to recruit respondents for health research quickly, but this strategy may yield participants who are less racially diverse, more educated, and older than the general population. Researchers should consider recruiting participants through other methods in addition to creating Facebook advertisements targeting underrepresented populations.

## Introduction

To improve health outcomes, researchers must engage in effective recruitment efforts to solicit large data pools of diverse populations for study participation [1]. However, an array of challenges has been noted in the literature, which may hinder these attempts. Specific facets of a study that may impact recruitment include study methodology, recruiter traits, insufficient respondent interest, and recruitment funding [2]. When attempting to attract vulnerable groups (ie, ethnic or racial minorities, those of low socioeconomic status, and residents of rural areas), recruitment has proven even more difficult owing to barriers such as respondent time constraints, reduced levels of health literacy, medical mistrust, and structural factors [3-6].

Consequently, during the COVID-19 pandemic, barriers and challenges of recruitment have expanded [7]. Namely, research activities have been halted or been modified from in-person studies to limit viral transmission, thus reducing participants’ interest in or capacity to continue their research engagement [7]. Moreover, financial recessions lead to heightened job loss and subsequent economic deprivation [8]. Therefore, we can assume that in uncertain times, individuals are inclined to focus on tasks that strengthen their day-to-day livelihood rather than health research participation.

Accordingly, the utilization of social media sites for health research data collection may lessen these obstacles. Generally, sites such as Facebook include daily users from various racial and ethnic backgrounds, levels of education and annual income, and geographic locations [9]. This approach also offers increased respondent convenience and volume completion with minimal staffing contribution [10]. In addition, the data collection modality allows participants to remain anonymous and maintain privacy throughout the process, which reduces their potential anxiety of direct research team interaction [11].

While reports of health research recruitment via social media have grown in recent years, the results of these efforts have been inconsistent [9,12]. Furthermore, social media has been used most often to recruit young adult populations for studies of substance abuse or sexual behavior. Less is known about how paid advertisements can be used to recruit participants for studies of specific health conditions such as breast health [9,12,13]. A study by Kapp et al [14] attempted to reach women aged 35-49 years from across the United States to complete a survey about breast cancer screening and was unable to recruit a single participant despite spending US $300 to field 3 advertisements over a 3-week period [14]. It seems reasonable that the effectiveness of using social media for research recruitment may be influenced by the characteristics of the study as well as the advertisement options used. As such, our study objectives were to describe the processes and evaluate the outcomes resulting from using Facebook advertisements to recruit a diverse sample of respondents to participate in a breast health study.

## Methods

### Eligibility

Participants were recruited to complete a Qualtrics survey about women’s knowledge of and communication with their mammography provider about breast density. Women, aged 40 years and older, who had not been diagnosed with cancer were eligible to participate. The survey tools consisted of 30 questions, and participants were not compensated. This study was approved by the University and Medical Center IRB at East Carolina University. Recruitment took place from June to July 2020.

### Advertisement Design

**Figure 1:**
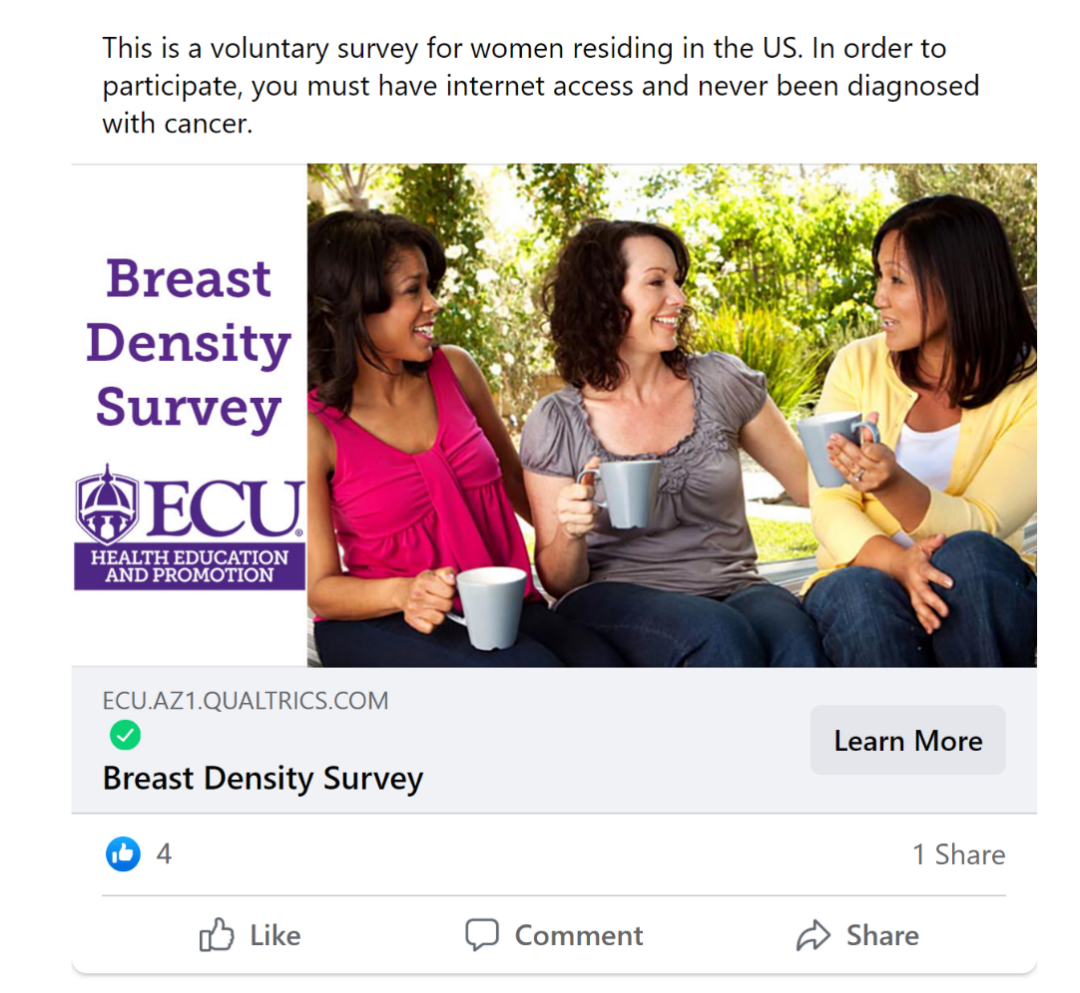
Sample advertisement.

Advertisements were purchased with US dollars and posted on Facebook and Instagram (Figure 1). The first round of advertisements ran from June 24 to July 1, 2020, and consisted of 45 individual advertisements. Each advertisement was assigned an identification number based on the combination of headings A-E and interest term groupings 1-9 used in this study (Table 1).

The headlines included phrases describing the eligibility criteria or survey topic. Interest terms allow researchers to find their target audience on the basis of the interests selected on the individual’s profile and other Facebook pages. Interest terms used during round 1 included topics such as health, cancer, and family. Round 1 included 5 different sets of advertisements; each set of advertisements used a different headline and interchanged the same 9 combinations of interest terms (Table 1).

The second round of advertisements ran from July 9 to July 16, 2020, and consisted of 10 individual advertisements. All round 2 advertisements used the same headline and interest terms found to be most impactful from among those in the first round of advertisements. In round 2, we focused on increasing the recruitment of ethnic and racial minorities to enhance the diversity of our sample. New interest terms related to ethnicity and behavioral targets were included in round 2. Behavioral targeting is based on a person’s activity on Facebook, including their purchase habits, travel activities, and internet browser usage. The behaviors selected for this advertisement set included multicultural affinities including African American and Asian American (Table 2).

**Table 1:** Headlines, interest terms, and advertisement IDs used in round 1.

Interest terms	Advertisement ID				
	Breast Density Survey	Voluntary For Women +40	Breast Density Awareness	Breast Density Knowledge	Women’s Health Research Survey
None	A1	B1	C1	D1	E1
Health	A2	B2	C2	D2	E2
Family	A3	B3	C3	D3	E3
Fitness and wellness	A4	B4	C4	D4	E4
Cancer awareness	A5	B5	C5	D5	E5
Women’s health	A6	B6	C6	D6	E6
Fitness and wellness, women’s health	A7	B7	C7	D7	E7
Health and beauty	A8	B8	C8	D8	E8
Fitness and wellness, family, health, cancer awareness, women’s health, and health and beauty	A9	B9	C9	D9	E9

**Table 2:** Interest terms, behavior terms, and advertisement IDs used in round 2.

Interest terms	Behavior terms	Advertisement ID
N/A^a^	N/A	AA1
N/A	Multicultural affinity: African American and Asian American	AA2
African American culture, African American history, Latino culture, Native American culture, Native American culture in the United States, and Asian American culture	N/A	AA3
African American culture, African American history, Latino culture, Native American culture, Native American culture in the United States, and Asian American culture	Multicultural affinity: African American and Asian	AA4
Family, African American culture, African American history, Latino culture, Native American culture, Native American culture in the United States, and Asian American culture	N/A	AA5
Family, African American culture, African American history, Latino culture, Native American culture, Native American culture in the United States, and Asian American culture	Multicultural affinity: African American and Asian American	AA6
Cancer awareness, African American culture, African American history, Latino culture, Native American culture, Native American culture in the United States, and Asian American culture	N/A	AA7
Cancer awareness, African American culture, African American history, Latino culture, Native American culture, Native American culture in the United States, and Asian American culture	Multicultural affinity: African American and Asian American	AA8
Fitness & wellness, African American culture, African American history, Latino culture, Native American culture, Native American culture in the United States, Asian American culture	N/A	AA9

^a^N/A: not applicable.

### Evaluation Metrics

Facebook advertising metrics, such as unique link clinks and cost per link click, were reviewed after each round. Unique link clicks measure how many people clicked on a link using a sampled portion of the data. Cost per link click measures the amount spent per link click [15]. These variables were analyzed to determine which advertisements were the most effective in terms of survey participants and cost per advertisement. All cost data is presented using US currency.

To optimize the budget for round 1 and our goal of generating cost-effective advertisements for round 2, we selected the lowest cost bid strategy. Many advertisements are often competing for the same individuals as they are members of multiple audiences [16]. Facebook uses auctions to determine which advertisement to show to a given user [16]. All advertisements that share target audiences must bid in an auction to be shown to a specific user. Facebook’s bid strategy allows advertisements to have the highest reach based on the goals and the budget set for the advertisements [17]. The lowest cost is a bid strategy that allows advertisement placement to be maximized by using the advertising budget to obtain maximum results or placements [18].

The second round of advertisements used cost cap as the bid strategy. Cost cap allows Facebook to determine how high or how low to bid to maximize the results of the advertisement without exceeding the stipulated cost cap [19]. The cost cap was $1 for round 1 and $4 for round 2. The daily budget limit for our first round of ads was $1 per day and $4 per day for round 2. This meant that Facebook could not spend more than that daily budget limit for each advertisement on a single day. Reach is the number of unique accounts that viewed an advertisement [20]. Overall and age group–stratified reach statistics were reviewed for each advertisement. Placement reach was also analyzed for advertisements posted on both Facebook and Instagram.

## Results

### Advertisement Performance

**Figure 2:**
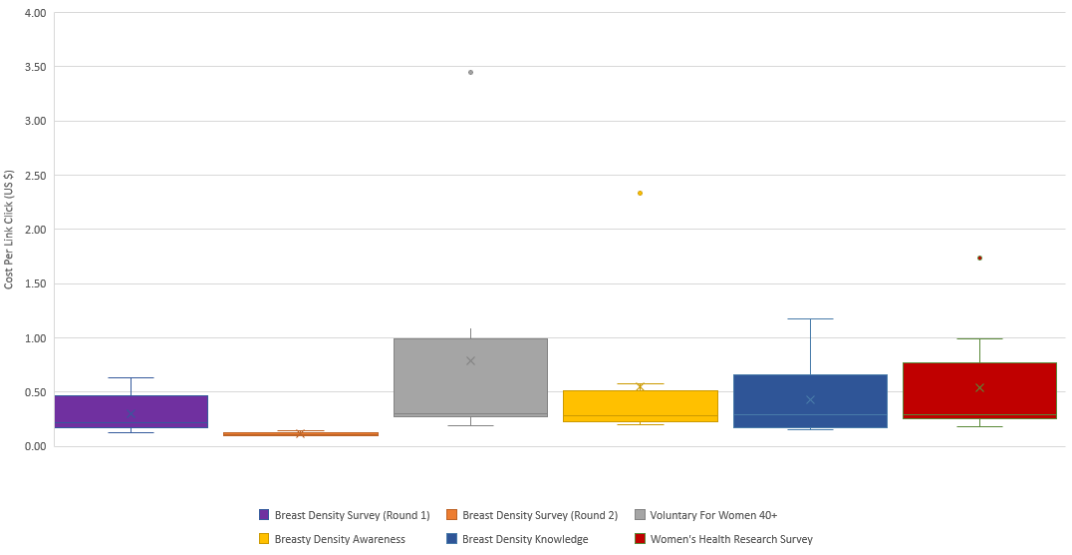
Facebook Recruitment Cost Statistics.

Despite advertisements being posted on Instagram, virtually all of the participants across both rounds were recruited through Facebook, with 97.4%% of link clicks coming from Facebook. Costs statistics for both rounds are displayed in Figure 2.

For round 1, the headline *Breast Density Survey* had the highest number of link clicks (n=148), and the headline *Voluntary For Women 40+* had the fewest link clicks (n=74). The best-performing advertisement in this round was A7, which had the *Breast Density Survey* headline. Advertisement A7 generated 28 unique link clicks at $0.12 per link click. *Breast Density Survey* was the best-performing headline in round 1, with an average cost per link click of $0.30. The worst-performing headline for round 1 was *Voluntary for Women 40+.* This headline had the lowest average unique link click (8) and the most expensive average cost per link click (US $0.79). Additionally, advertisement B7, which combined this headline and all of the interest terms, resulted in 2 unique link clicks at $3.45 per link.

The best-performing headline from round 1, *Breast Density Survey*, was used for all round 2 advertisements. The total average unique link clicks in round 2 was 108, and the average cost per link click was $0.11. Advertisements AA7 and AA9, which did not contain behavioral terms, generated the highest number of link clicks in round 2 (n=124). However, with one exception, the advertisements which contained behavioral terms AA4, AA6, and AA8 were the most cost-effective at $0.10 per unique link click.

### Respondent Demographics

In round 1, a total of 179 people clicked on the Qualtrics survey link, of whom 164 (91.62%) were eligible to complete the survey (Table 3).

The majority of women recruited were in the age range of 55-64 years or 65 years and older, with each group accounting for 34.33% of respondents. In total, 78% of respondents were White and almost two-thirds (57.43%) had a college or graduate degree. In round 2, a total of 478 people clicked on the survey link, and 418 (87.45%) participants were eligible to complete the survey. The largest group of women recruited were in the 55-64–year age range, representing 36.72% of the participants, and were White, accounting for 81.34% of participants. Over two-thirds (66.21%) of the women recruited during this round had a college education or more.

Across both rounds, we reached 50,017 unique accounts, 1693 link clicks were generated, 657 people accessed the survey, and 582 women were eligible to complete the entire survey. This resulted in a study conversion rate of 3.38%. The conversion rate (defined as the number of link clicks divided by the number of individuals reached, multiplied by 100) is another measurement of advertisement effectiveness. The survey’s participation rate (the number of participants starting the survey divided by reach) was 1.31%, and the eligibility rate was 88.58%. Over half of the eligible respondents were over the age of 55 years (63.94%), and the majority identified as White (80.41%) and had a college degree or higher (63.71%).

**Table 3:** Survey access and eligibility statistics.

Headlines			Survey link clicks by women aged 40-44 years, n (%)		Survey link clicks by women aged 45-54 years, n (%)		Survey link clicks by women aged 55-64 years, n (%)		Survey link clicks by women aged 65 years and older, n (%)		Total link clicks, n (%)		Qualtrics survey accessed, n			Total eligible individuals, n (%)	
**Round 1**														179			164 (91.62)
	Breast Density Survey	9 (8.27)		27 (18.35)		55 (34.11)		57 (39.27)		148 (100)							
	Voluntary For Women 40+	5 (4.90)		13 (16.50)		28 (41.19)		28 (37.41)		74 (100)							
	Breast Density Awareness	4 (4.24)		22 (22.86)		34 (29.33)		50 (43.57)		110 (100)							
	Breast Density Knowledge	5 (2.99)		19 (13.64)		38 (32.11)		62 (51.26)		124 (100)							
	Women’s Health Research Survey	10 (7.21)		19 (18.68)		29 (27.71)		49 (46.40)		107 (100)							
**Round 2**														478			418 (87.45)
	Breast Density Survey	91 (7.96)		273 (24.34)		437 (38.85)		329 (28.86)		1130 (100)							

## Discussion

### Summary of Results

Given the variety of existing social media platforms, we sought to understand how Facebook advertisements could be leveraged to recruit respondents for a survey on breast health. We found Facebook advertisements to be an efficient and effective recruitment tool. Two rounds of Facebook advertisements were fielded over 2 weeks to determine what combinations of advertisements would be the most cost-effective and yield a diverse survey sample. By the final round, we were able to produce advertisements that averaged $0.11 per link click, a conversation rate of 3.38%, and had a study eligibility rate of 88.58%. We found that advertisements using study-specific headlines (ie, *Breast Density Survey*) and health-related interest terms were most successful.

### Comparison With Other Studies Involving Social Media Recruitment

Our advertisements performed better than those in the majority of studies included in Whitaker et al’s [9] systematic review that evaluated the performance of Facebook advertisements used to recruit participants for health research. Our final cost per link click value was $0.11, compared to an average of $0.51 per link click across included studies [9]. Additionally, our advertisements ran for a shorter period of time and reached fewer devices but resulted in higher eligibility rates and lower recruitment costs per eligible participant than the averages reported by Whitaker et al [9] and other recent studies [13]. Additionally, the studies included in Whitaker et al’s [9] review focused health issues such as drug use, sexual health, and pregnancy in young adult populations. Ours is one of the few studies evaluating the use of Facebook advertisements to recruit middle-aged and older adults for health research [9].

Kapp et al [14] is one of the few accounts describing the use of Facebook advertisements to recruit middle-aged women to assess breast cancer screening beliefs. This study did not recruit any participants through Facebook despite fielding advertisements for a similar number of weeks. However, there are notable differences between both studies. Our study was conducted in 2020, while Kapp et al [14] recruited during 2012, during which time social media usage has grown. In total, 53% of adults reported using any type of social media platform in 2012 compared to 72% in 2020 [21]. In the same time frame, Facebook usage has increased the most for middle-aged and older adults in the United States, leading to a larger eligible population [22,23]. Kapp et al [14] did not provide information about advertisement characteristics such as the use of interest terms, behavioral targets, or bid strategies. These options were likely not available at the time of the study and are missing from many recent descriptions of social media recruitment [9,12].

Another important consideration is the timing of the data collection. We placed our advertisements during the summer of 2020, a time when a larger percentage of the public stayed home as a result of the COVID-19 pandemic, which may have led to more favorable outcomes. Ali et al [24] used Facebook advertisements to survey adults in the United States about COVID-19 beliefs and behaviors. Ali et al [24] fielded their survey between March 20 and March 30, 2020, at a time when most of the population were subject to stay-at-home orders. Due to both a more expansive target population of all adults and the timing of the advertisements, Ali et al [24] generated a wider reach than our study (ie, 236,017 vs 50,017). However, our outcomes of cost per link click ($0.09 vs $0.11), conversation (4.1% vs 3.88%), and eligibility rates (99.4% vs 88.58%) were comparable. These data suggest that the increase in internet activity continued through the summer months despite many locations loosening COVID-19–related restrictions and warmer weather, allowing people to spend more time outdoors. According to the Pew Research Center, Facebook usage in early 2021 remains at 2020 levels [25]. If these trends persist, Facebook advertisements may be an increasingly important and cost-effective way to recruit research participants, but this option is not without its challenges.

Despite the higher percentage of Black and Latinx populations reporting Facebook usage, our first round of survey responses was predominately White (78.05%) [21]. Given that it was not possible to limit advertisement audiences by race, we added interest terms mentioning cultural interests of communities of color and similar behavioral targets to the round 2 advertisements. The individual percentages of all racial or ethnic groups (ie, White women and women of color) rose in round 2, while the percentage of women listing their race as “Other” declined. While culturally specific interest or behavioral terms are assumed to capture mostly women of color, there are likely White women with these interests on their profiles, thus diluting the potential gains in recruitment of women of color. Ali et al [24] described similar challenges and solutions with respect to recruiting a diverse sample but was not able to report the effectiveness of their strategies as they only posted the revised advertisements for 1 day.

While gains in racial diversity in round 2 respondents were limited, the use of culturally specific terms improved the age distribution of respondents with a larger percentage of women under the age of 65 years being recruited. This is likely owing to the fact that populations of color tend to be younger than White populations; hence, efforts to increase racial diversity also generated a younger sample. Facebook has relatively older users than other social media platforms, those in the age range of 30-49 years report using Facebook the most (77%), followed by those aged 50-64 years (73%), compared to only 50% of those aged 65 years and older [25]. The improved age distribution brings the sample more in line with the demographics of Facebook users, but it is unclear how other types of diversity such as sexual orientation or ability status can be addressed during recruitment.

### Limitations

Study findings should be viewed in the context of the following limitations. First, the COVD-19 pandemic has influenced the number and types of potential survey respondents available. Given that individuals with higher education levels and incomes are more likely to work from home; those same individuals had more opportunities to be on the internet and on social media. As a result, participants recruited during that time were more likely to be of higher socioeconomic status. Additionally, our ability to target specific populations relies on the accuracy of Facebook data. Recent reports indicated that users who accessed the platform more frequently or those with older profiles had more categories listed on their advertisement preference pages and reported these categories with increased accuracy [26]. This might lead to the recruitment of heavier users or earlier adopters of social media. These groups might have different attitudes and beliefs related to the health issue being assessed.

### Conclusions

Our study offers complete information about the development and success of different advertisements and cost strategies, which can help researchers target specific populations for recruitment [9]. A detailed description of a process for developing the most cost-effective advertisement targeting middle-aged adults was described. Considering increased social media usage, these approaches can support health research and accelerate recruitment goals. However, challenges related to achieving a diverse study population were detected. While current approaches do not address all diversity-related concerns, this study provides data that help guide new strategies to create a well-balanced sample.
